# Unraveling the relationship between ACTH and cortisol levels in COVID-19 infections: A meta-analysis

**DOI:** 10.1371/journal.pone.0296281

**Published:** 2023-12-28

**Authors:** Zixin Cai, Bilian Liu

**Affiliations:** National Clinical Research Center for Metabolic Diseases, Key Laboratory of Cardiometabolic Medicine of Hunan Province, Metabolic Syndrome Research Center, The Second Xiangya Hospital of Central South University, Changsha, Hunan, China; King Abdulaziz University Faculty of Medicine, SAUDI ARABIA

## Abstract

**Objective:**

In December 2019, a novel pneumonia associated with the 2019 coronavirus emerged unexpectedly. However, limited data exist on the effects of COVID-19 on ACTH and cortisol levels. To address this gap in knowledge, we conducted a meta-analysis of published studies on the relationship between COVID-19 patients and their ACTH and cortisol levels.

**Methods:**

We conducted a thorough search of the PubMed, Embase, Cochrane Library, and Web of Science databases up until May 2023. We assessed the relevance of each study we found, specifically looking for studies that reported on ACTH and cortisol levels in COVID-19 patients. We calculated weighted mean differences (WMD) and 95% confidence intervals (CI) to investigate the relationship between ACTH and cortisol levels in COVID-19 patients. We evaluated the quality of each study using the Newcastle Ottawa scale (NOS), and we assessed publication bias using Begg’s rank correlation test, Egger’s test, and funnel plot. We conducted our meta-analysis using the Stata 12.0 (Stata Corporation, TX).

**Results:**

Our search yielded nine studies that met our inclusion criteria, which included a total of 440 COVID-19 patients and 474 controls, with data up to May 2023. Seven of these studies reported on ACTH levels, and six studies reported on cortisol levels. Our findings revealed that COVID-19 patients had significantly higher levels of cortisol compared to controls (WMD 3.46 (95% CI 2.29 to 4.62)). However, there was no significant difference in ACTH levels between COVID-19 patients and controls (WMD 1.58 (95% CI -5.79 to 8.94)).

**Conclusions:**

This meta-analysis indicates a potential relationship between elevated cortisol levels and COVID-19 infection. However, more well-designed, adequately powered, randomized controlled trial will be needed to assess the use of cortisol in patients with COVID-19 infection.

## Introduction

In March 2020, the World Health Organization (WHO) officially declared the COVID-19 outbreak a global pandemic, marking the rapid spread of the virus across continents. By early 2023, the WHO reported approximately 750 million confirmed cases of COVID-19 and over 6.8 million deaths worldwide. The global death rate for this disease is estimated at about 5.8%. The primary symptoms of COVID-19 include pneumonia, fever, muscle pain (myalgia), and fatigue [1]. This pandemic has inflicted significant physical, psychological, and economic strains on individuals, healthcare systems, and economies around the globe.

Adrenal insufficiency is a serious, potentially life-threatening condition arising from inadequate adrenal cortisol production. This insufficiency can be due to primary adrenal insufficiency (PAI), where the adrenal glands themselves are diseased, or secondary adrenal insufficiency (SAI), resulting from a deficiency of adrenocorticotropic hormone (ACTH) [2, 3]. Chronic hypothalamic-pituitary-adrenal (HPA) insufficiency, characterized by HPA axis impairment, is another aspect of this condition. The HPA axis plays a vital role in regulating cortisol production in humans. Early diagnosis and prompt treatment are essential, as timely hormonal replacement therapy can be lifesaving [3]. Despite early diagnosis and comprehensive treatment, mortality rates for adrenal insufficiency remain relatively high [4, 5], and patients experience decreased quality of life (QOL) [6, 7] and increased risk of adrenal crisis [8, 9]. Adrenal insufficiency can present with nonspecific or asymptomatic symptoms, leading to potential diagnostic delays, with only 15% of patients receiving a correct diagnosis during the initial medical encounter [10]. Once adrenal insufficiency is suspected, biochemical testing is needed to help identify the diagnosis [2]. Biochemical testing is crucial for confirming a diagnosis of adrenal insufficiency, with baseline morning serum cortisol levels and ACTH stimulation tests being key initial steps in the evaluation process.

Prior research has explored the impact of COVID-19 infection on cortisol and ACTH levels, yet the findings have been varied and inconclusive. To address this, we undertook a meta-analysis, reviewing all published studies concerning this subject. Our goal is to offer a thorough and unbiased evaluation of how COVID-19 infection influences cortisol and ACTH levels. This analysis aims to resolve the discrepancies observed in earlier studies and to furnish evidence-based recommendations that will inform future clinical practices and research in this field.

## Method

### Search strategy

This systematic review was conducted in accordance with the PRISMA guidelines (Preferred Reporting Items for Systematic Reviews and Meta-Analysis) (S1 Checklist. PRISMA 2020 checklist.) [11]. We searched multiple databases including PubMed, Web of Science, Embase, and Cochrane Library, without any language restrictions, from their earliest inception until May 2023. Our search strategy included the use of keywords and Medical Subject Headings (MeSH) terms related to "COVID-19" or "SARS-CoV-2", "ACTH", and "cortisol". In addition, we conducted manual cross-referencing of the included articles to supplement the electronic search. The full-text articles were independently reviewed and duplicates were eliminated to ensure eligibility. Any discrepancies between reviewers were resolved through consensus. By following the PRISMA guidelines, we aimed to ensure the quality and transparency of our systematic review and meta-analysis.

### Study selection

The inclusion criteria for this study are as follows: (1) observational studies (cohort, case-control, and cross-sectional studies) conducted on humans, (2) focused on COVID-19, and (3) evaluating serum biochemical outcomes such as ACTH and cortisol. Studies that did not report individual or mean serum levels of ACTH and cortisol were excluded, as were those that involved the concurrent use of other steroids. Additionally, abstracts, commentary articles, conference posters, reviews, letters to the editor, and editorials were excluded from the analysis.

### PICO framework

1. Population (P): The population in our study consisted of individuals infected with COVID-19, irrespective of their age, gender, or disease severity.

2. Intervention (I): The main intervention considered in our study was the presence or measure of ACTH and cortisol in the patient’s system.

3. Comparison (C): We compared the individuals with different levels of ACTH and cortisol and their associated COVID-19 outcomes.

4. Outcome (O): The primary outcomes measured were the levels of ACTH and cortisol.

### Data abstraction and quality assessment

The data extraction for our study was meticulously performed by two independent researchers using a standardized form. This form captured key details such as the first author’s name, date of publication, country of origin, study design (whether it was a cohort, case-control, or cross-sectional study), sample sizes for both cases and controls, and the specific indicators analyzed (ACTH or cortisol). Mean and standard deviation values were also extracted using these forms, a process independently executed by two reviewers, BL and ZC.

To evaluate the quality of the selected studies, we employed the Newcastle-Ottawa Quality Assessment Scale (NOS) [12]. This scale assesses each study across three dimensions: selection, comparability, and exposure, with a total possible score ranging from 0 to 9. Studies achieving NOS scores of 6 or higher were deemed to be of high quality [12]. The NOS score of each study is presented in Table 1.

**Table 1:** Studies included in the meta-analysis.

Number	Author	Country	Year	Sutdy design	Indicators	COVID-19	Control	NOS
**1**	**Iraj Ahmadi**	**Iran**	**2022**	**Cohort study**	**Cortisol/ACTH**	**14**	**140**	**7**
**2**	**Wei Ting Gu**	**China**	**2021**	**Case-control study**	**Cortisol/ACTH**	**43**	**45**	**8**
**3**	**Emre Urhan**	**Turkey**	**2022**	**Cohort study**	**ACTH**	**43**	**11**	**7**
**4**	**Mustafa Sait Gonen**	**Turkey**	**2022**	**Case-control study**	**Cortisol/ACTH**	**49**	**28**	**8**
**5**	**Maria P. Yavropoulou**	**Greece**	**2022**	**Case-control study**	**ACTH**	**52**	**33**	**8**
**6**	**I. Ekinci**	**Turkey**	**2021**	**Case-control study**	**Cortisol/ACTH**	**74**	**33**	**6**
**7**	**Bharat Kumar**	**India**	**2021**	**Cohort study**	**Cortisol**	**126**	**109**	**9**
**8**	**Liza Das**	**India**	**2021**	**Cross-Sectional**	**Cortisol/ACTH**	**35**	**49**	**7**

### Statistical analysis

For our meta-analysis, we utilized STATA version 12.0 software (StataCorp, Texas, USA). We analyzed outcomes related to ACTH or cortisol using the weighted mean difference (WMD) method, along with a 95% confidence interval (CI). When data were presented as mean and standard error of the mean (SEM), we calculated the standard deviation by multiplying the SEM by the square root of the sample size. To identify heterogeneity in the studies, we applied the I square (*I*^*2*^) test, defining significant heterogeneity as an *I*^*2*^ value greater than 50% accompanied by a *p*-value less than 0.05. Depending on the degree of heterogeneity detected, we conducted the meta-analysis using either a random-effect or a fixed-effect model. Specifically, in cases without significant heterogeneity, the fixed-effect model was employed, while the random-effect model was used in the presence of significant heterogeneity. To test the robustness of the results, sensitivity analysis was conducted by leave-one-out analysis for cortisol and ACTH. We considered *p*-values below 0.05 to be statistically significant.

## Results

### Study selection

**Fig 1:**
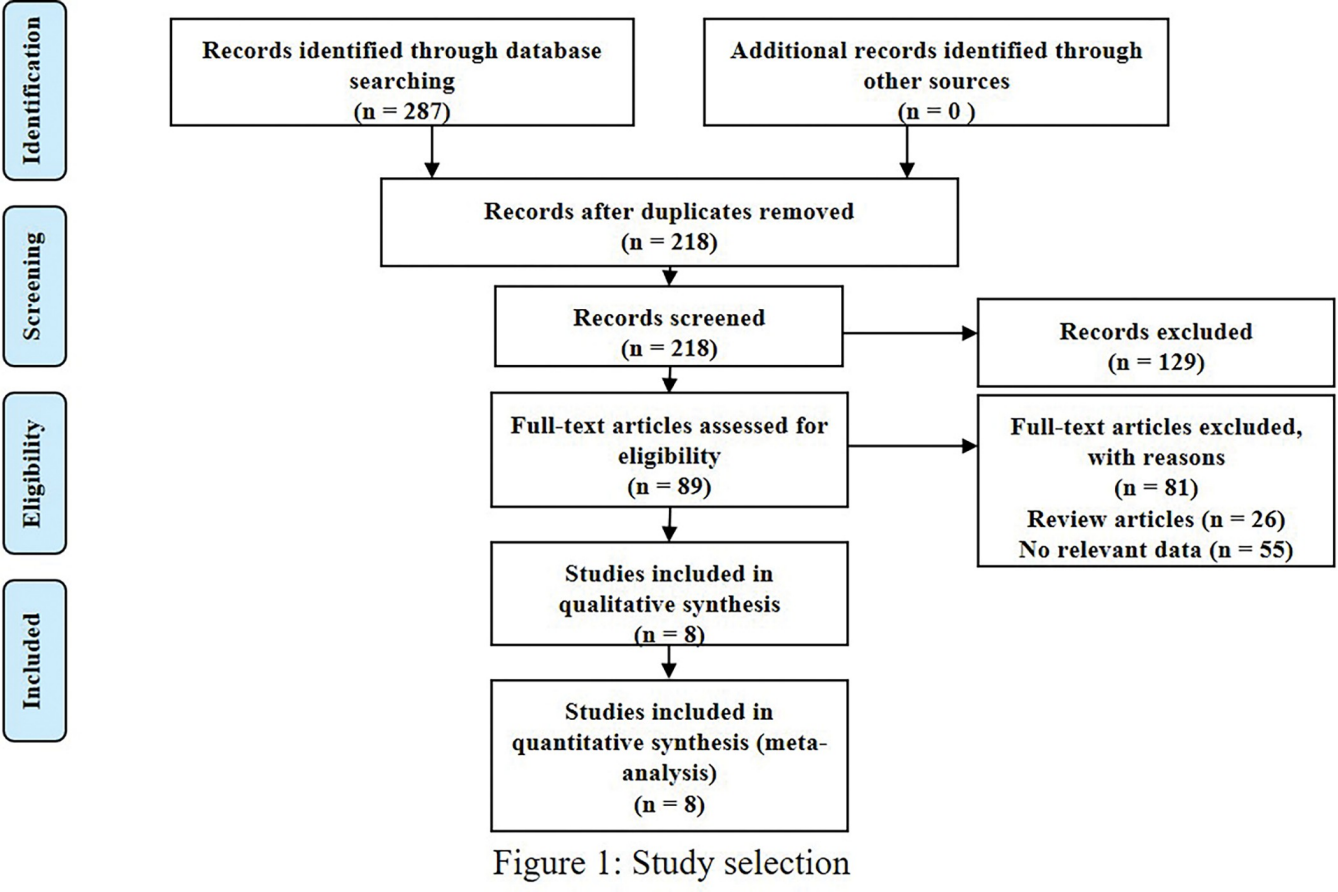
Flow chart of the study selection process.

We conducted an initial search across four databases and retrieved a total of 287 articles published up until May 2023 (as illustrated in Fig 1). Following the removal of duplicate articles, 158 papers were assessed based on their titles and abstracts. The full text of the remaining 90 articles was then reviewed for eligibility for inclusion in our meta-analyses, resulting in the exclusion of 81 articles for reasons such as being case reports, abstracts, letters, or editorials. Finally, nine studies that met our inclusion criteria were selected for analysis, and Table 1 summarizes the key characteristics of these studies [13–21]. Fig 1 provides a visual depiction of the number of articles included and excluded at each stage of our evaluation process, together with the reasons why certain studies were excluded. Overall, the combination of Fig 1 and Table 1 provides readers with a comprehensive understanding of our evaluation process and the studies that we ultimately included in our meta-analyses.

### Description of the included studies

This meta-analysis comprised nine articles, involving a total of 914 patients. The sample sizes of the included studies ranged from 30 to 235 subjects, and their quality ratings ranged from six to nine stars. Of the included studies, seven evaluated patients with ACTH, while six evaluated patients with cortisol. Table 1 presents a concise summary of the key features of each of the studies included in this meta-analysis. All of the studies were published between 2020 and 2022 and consisted of three cohort studies, four case-control studies, and two cross-sectional study. These studies were conducted in diverse geographic locations, with three from Turkey, two from India, Iran and one each from China, and Greece.

### Overall analysis

**Fig 2:**
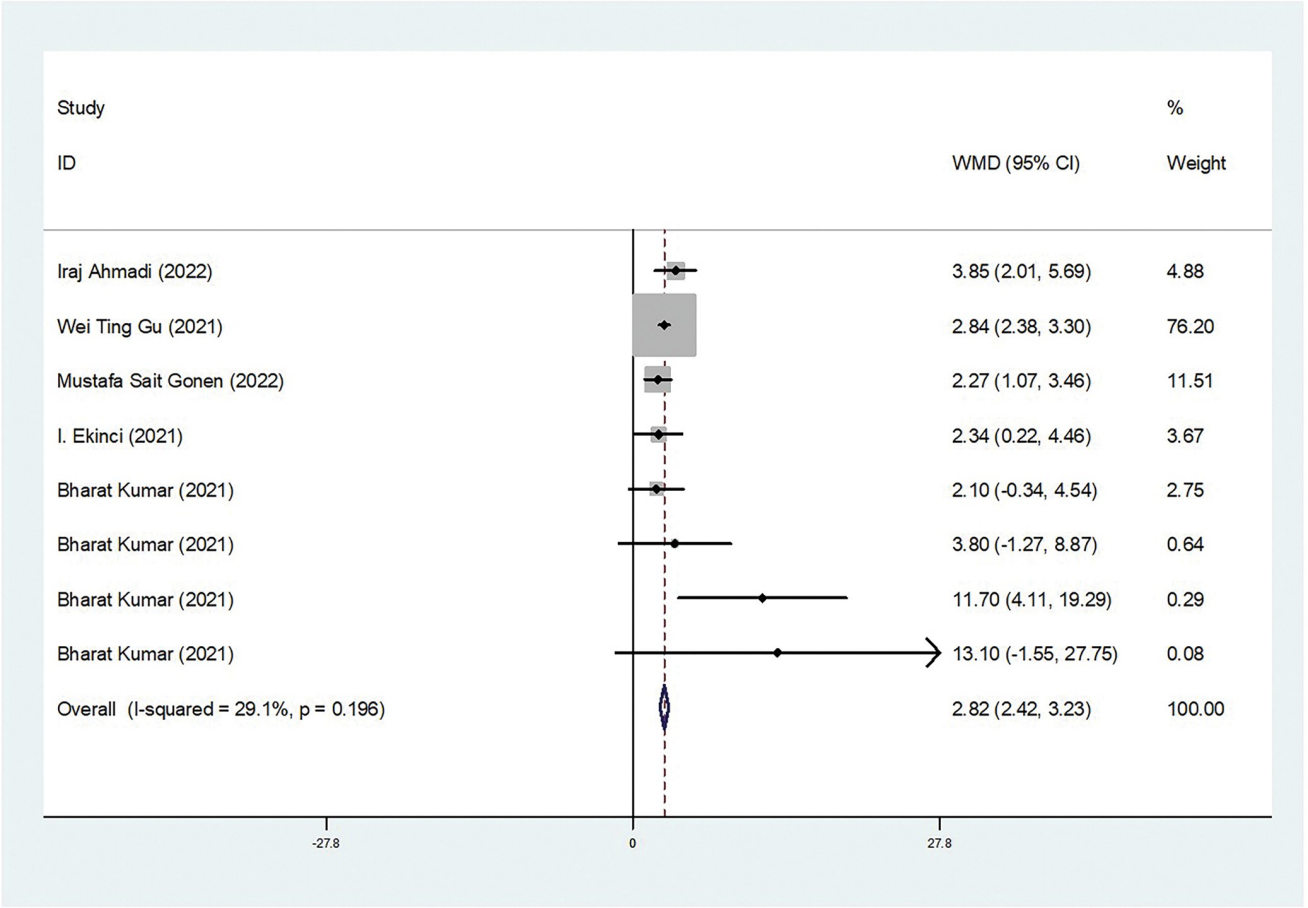
Forest plot for the WMD of cortisol levels between the COVID-19 and control groups.

**Fig 3:**
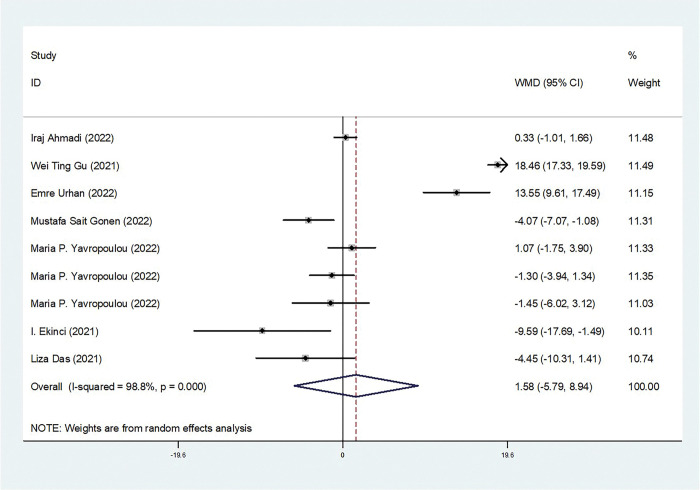
Forest plot for the WMD of ACTH levels between the COVID-19 and control groups.

**Fig 4:**
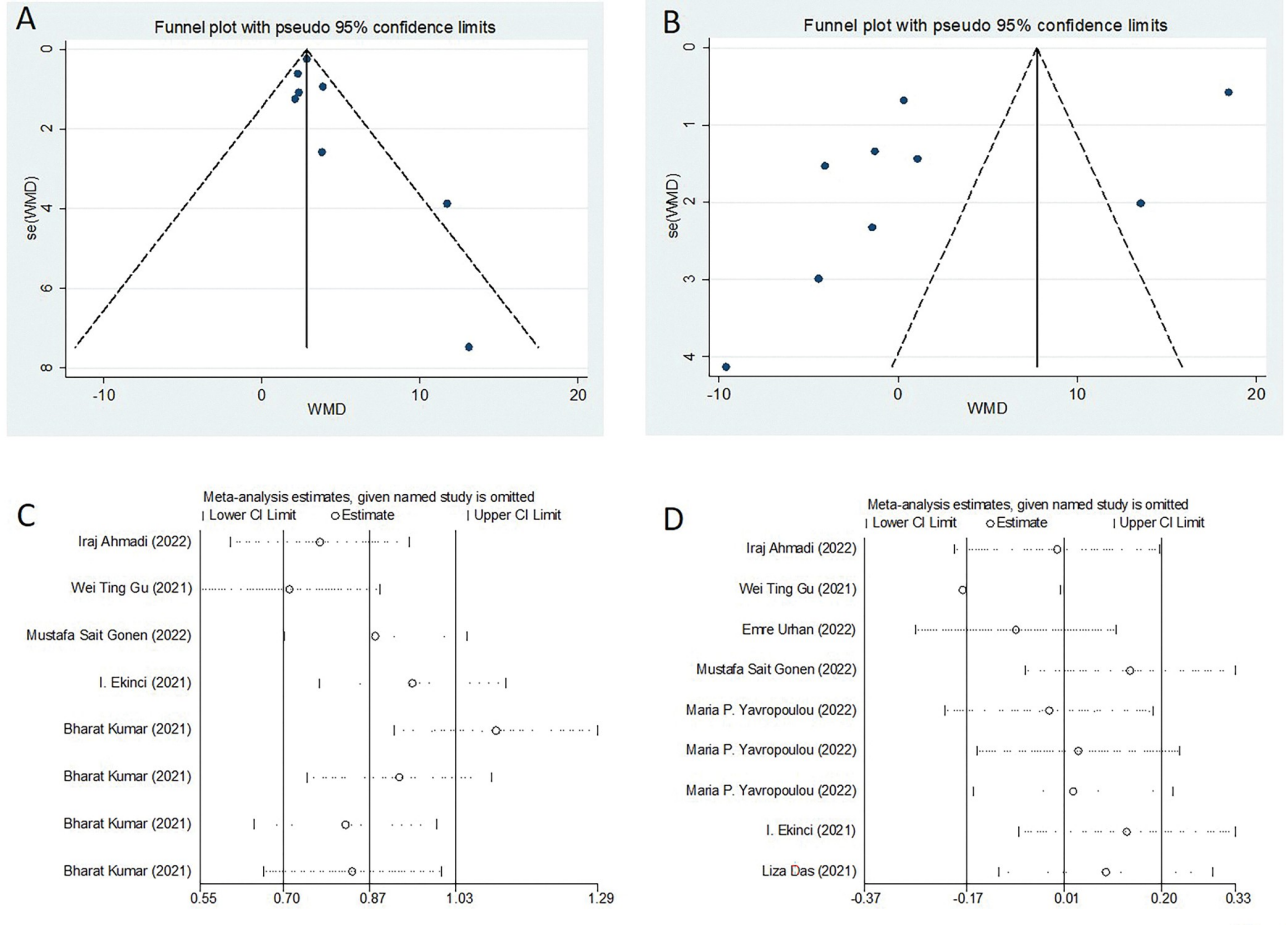
Sensitivity analysis and publication bias funnel plots.

Our meta-analysis revealed a statistically significant increase in serum cortisol concentrations among COVID-19 patients compared to controls (weighted mean difference [WMD]: 3.46; 95% confidence interval [CI]: 2.29 to 4.62) (Fig 2). The studies included in the analysis show heterogeneity (*I*^*2*^ = 62.6%). Regarding serum ACTH concentrations, our overall analysis did not demonstrate a statistically significant difference between COVID-19 patients and controls (WMD: 1.58; 95% CI: -5.79 to 8.94) (Fig 3). The significance of these findings may suggest that COVID-19 patients experience a dysregulation in cortisol levels, which could potentially contribute to the pathophysiology and clinical manifestations of the disease. We also conducted tests for publication bias, including Begger’s test, Egger’s test (data not shown), and funnel plot analysis, and all the tests indicated the absence of publication bias (Fig 4A and 4B). Additionally, the sensitivity analyses we performed indicated the robustness of our conclusions (Fig 4C and 4D). Our findings suggest that serum cortisol levels may be elevated in COVID-19 patients, while serum ACTH concentrations may not be significantly affected. These results were consistent across different sensitivity analyses and publication bias tests, further supporting their validity.

## Discussion

### Association between ACTH and cortisol levels and COVID-19

To the best of our knowledge, this study represents the first comprehensive meta-analysis investigating the association of ACTH and cortisol levels with COVID-19 infection in humans. We identified and analyzed 9 relevant studies, which collectively included 914 patients of all ages. Our findings indicate a significant association between elevated cortisol levels, suggesting a possible mechanism underlying COVID-19 infection-induced risk in the infected patients. This is consistent with previous finding that cortisol is used for acute respiratory infections **[22]**.

In the context of the growing evidence surrounding hormonal disturbances in COVID-19, the study by Ramezani et al. (2020) is particularly noteworthy [21]. Their research concentrated on the relationship between anxiety, cortisol levels, and COVID-19 outcomes. They discovered that patients who died from COVID-19 had significantly higher cortisol levels compared to those who recovered. Additionally, their findings revealed a positive correlation between cortisol levels and scores on the Hospital Anxiety and Depression Scale (HADS), suggesting that stress and anxiety might induce higher cortisol levels, which in turn could serve as a prognostic marker in COVID-19. This study sheds light on the intricate connection between psychological factors and physiological responses in the course of COVID-19. The stress-induced activation of the hypothalamic-pituitary-adrenal (HPA) axis, resulting in elevated cortisol levels, could potentially worsen inflammatory responses and affect the severity of the disease. These findings underscore the importance of incorporating psychological interventions and stress management into the comprehensive care of COVID-19 patients.

### Underlying mechanisms of COVID-19 effects on ACTH and cortisol levels

Firstly, stress levels can significantly impact metabolic, cardiovascular, and immune systems by elevating cortisol levels. Additionally, under stressful conditions, cortisol metabolism and cortisol-binding globulin (CBG) metabolism can be inhibited, leading to increased cortisol activity [23]. Additionally, elevated cortisol levels in COVID-19 patients could also be attributed to heightened stress and fear of mortality related to the pandemic [24].

Secondly, elevated cortisol levels in COVID-19 patients could be attributed to proinflammatory cytokine IL-6. Previous studies have suggested that IL-6 acts like an ACTH-like stimulus for the glucocorticoid-secreting adrenal fasciculus [25], which could result in increased cortisol secretion under prolonged stressful circumstances [26]. Researchers have also identified the expression of IL-6 and IL-6 receptors in primary cultures of human adrenal cells deficient in macrophages (CD68-positive cells), mainly in the reticular zone but also within the fascicular zone and in individual cells in the glomerular zone and medulla [27].

Our analysis found no significant differences in the circulating levels of ACTH between COVID-19 patients and controls. Interestingly, some researchers have proposed an intriguing hypothesis that suggests SARS-CoV expresses an amino acid sequence that shares molecular homology with ACTH. As a result, it may block the stress-induced cortisol response in the host by producing antibodies against ACTH [28]. These variations might be attributed to the virus’s direct impact on the HPA axis or secondary effects due to systemic inflammation [29, 30]. Such findings suggest a complex interplay between the virus and the endocrine system, impacting both the course of the disease and the patient’s response to treatment. Therefore, a nuanced understanding of ACTH levels and their implications in COVID-19 is crucial for developing effective therapeutic strategies and managing patient care more effectively.

However, more research is needed to confirm these hypotheses and gain a better understanding of the mechanisms underlying COVID-19’s effects on ACTH and cortisol levels.

### Strengths and limitations

Although our meta-analysis presents significant findings, it’s important to acknowledge its potential limitations. Firstly, the inclusion of studies using varied cortisol assays could have influenced our results due to the heterogeneity of testing methods. Secondly, significant heterogeneity was observed among the studies. While we employed a random-effects model to account for this variability, it’s possible that not all differences between studies were fully explained. Thirdly, our analysis was based solely on observational studies, which limits our ability to establish causality in the relationship between cortisol levels and COVID-19 infection. Finally, the patient data in our analysis was drawn from only five countries, potentially limiting the broader applicability of our findings to other ethnic and geographical groups.

Despite these limitations, our study stands as a pioneering effort in exploring the potential link between cortisol levels and COVID-19 infection risk. The insights gained from our analysis can pave the way for further research in this area and enhance our understanding of how HPA axis function may play a role in COVID-19 infection.

## Conclusion

In summary, there is a significant association between higher cortisol levels and subclinical COVID-19 infection, which could subsequently increase the risk of COVID-19 infrection. Therefore, it is crucial to take note of elevated cortisol levels in patients, as they may indicate a higher severity of COVID-19.

## Supporting information

S1 Checklist PRISMA 2020 checklist. (DOCX)
